# Supplementary material for: Influence of Different Types of β-Blockers on Mortality in Patients on Hemodialysis
Source: Biomedicines. 2023 Oct 19;11(10):2838. doi: 10.3390/biomedicines11102838 (PMC10603865; doi:10.3390/biomedicines11102838)
Supplement: Supplementary file 1 [file biomedicines-11-02838-s001.zip › Supplementary material/Supplementary materials.doc]

**Supplementary Material**

**Table S1.** Types of medications and Health Insurance Review and Assessment Service codes…….1

**Table S2.** ICD–10 codes used in Charlson Comorbidity Index........………………….……..……….6

**Table S3.** Codes associated with cardiac and cerebrovascular outcome.…………….....…….….7

**Figure S1.** Forest plots of the HR and 95% CI according to subgroups…………………………….…8

**Figure S2.** Kaplan–Meier curves depicting CVE-free survival based on different groups…………..10

**Table S4.** Cox regression analyses for mortality in patients with MI, CHF, or atrial fibrillation…….11

**Table S5.** Cox regression analyses for CVE……………………………………………………….….12

**Table S6** Summary of previous studies for the association between the type of β–blockers and clinical outcomes................................................................................................................................................13

**Table S1. Types of medications** and Health Insurance Review and Assessment Service codes

| **Medications** | **Codes** |
| --- | --- |
| **Acepril** | 104201ATB, 104202ATB |
| **Amlodipine** | 495901ATB, 459802ACH, 483201ATB, 486501ATB, 107601ATB, 107601ATD, 459801ACH, 459801ATB, 459901ATB, 464601ATB, 470801ATB, 476201ATB, 479701ATB, 483202ATB, 486502ATB, 107602ATB, 107602ATD, 470802ATB |
| **Amlodipine+Atorvastatin** | 614500ATB, 472300ATB472400ATB472500ATB518900ATB |
| **Amlodipine+Losartan+Chlorthalidone** | 662800ATB, 662900ATB, 663000ATB |
| **Amlodipine+Losartan+Rosuvastatin** | 663900ATB, 664000ATB, 664100ATB, 664200ATB, 664300ATB, 664400ATB |
| **Amlodipine+Olmesartan+Rosuvastatin** | 677300ATB, 677400ATB, 677500ATB, 677600ATB |
| **Amlodipine+Rosuvastatin** | 673900ATB, 674000ATB, 674100ATB |
| **Amlodipine+Rosuvastatin+Telmisartan** | 671200ATB, 671300ATB, 671400ATB, 671500ATB, 677000ATB, 677100ATB, 671600ATB, 671700ATB |
| **Amlodipine+Telmisartan+Hydrochlorothiazide** | 663500ATB, 663600ATB, 663700ATB, 663800ATB |
| **Amosulalol** | 107901ATB, 107902ATB |
| **Arotinolol** | 110202ATB, 110201ATB |
| **Atenolol** | 483102ATB, 111402ATB, 483101ATB, 111403ATB, 111401ATB |
| **Atenolol+Chlorthalidone** | 262100ATB, 460200ATB |
| **Azilsartan** | 662401ATB, 662403ATB, 662402ATB |
| **Azilsartan+Chlorthalidone** | 673500ATB, 673600ATB |
| **Barnidipine** | 114003ACH, 114001ACH, 114002ACH |
| **Benidipine** | 115101ATB, 115102ATB, 115104ATB, 115103ATB |
| **Betaxolol** | 116801ATB, 116803ATB |
| **Bevantolol** | 117002ATB, 117001ATB |
| **Bisoprolol** | 117904ATB, 117903ATB, 117902ATB, 117901ATB |
| **Bisoprolol+Hydrochlorothiazide** | 469800ATB, 470000ATB, 469900ATB |
| **Candesartan** | 122601ATB, 122602ATB, 122603ATB |
| **Candesartan+Amlodipine** | 652900ATB, 653000ATB, 653100ATB |
| **Candesartan+Hydrochlorothiazide** | 423700ATB |
| **Candesartan+Rosuvastatin** | 661800ATB, 661900ATB, 673700ATB, 662000ATB, 662100ATB |
| **Captopril** | 122901ATB, 122902ATB, 122903ATB |
| **Captopril+Hydrochlorothiazide** | 262200ATB, 262300ATB |
| **Carteolol** | 124801ATB |
| **Carvedilol** | 125005ATB, 125003ATB, 662201ATB, 125008ACR, 125001ATB, 662202ATB, 125007ACR, 125002ATB, 125006ACR, 125004ACR |
| **Celiprolol** | 129101ATB |
| **Cilazapril** | 133001ATB, 133002ATB, 133003ATB |
| **Cilnidipine** | 133102ATB, 133101ATB |
| **Clonidine** | 136505ATR |
| **Diltiazem** | 145706ATB, 145707ACR, 145707ATR, 145703ACR, 145706ATR, 145707ATB |
| **Doxazocin** | 149101ATB, 149102ATB, 149104ATR, 149103ATB |
| **Efonidipine** | 441202ATB, 441201ATB |
| **Enalapril** | 151603ATB, 151601ATB |
| **Enalapril+Hydrochlorothiazide** | 453700ATB, 440300ATB |
| **Eprosartan** | 429201ATB |
| **Eprosartan+Hydrochlorothiazide** | 460500ATB |
| **Felodipine** | 157503ATR, 157501ATR |
| **Felodipine+Metoprolol** | 262400ATR |
| **Fimasartan** | 515203ATB, 515201ATB, 515202ATB |
| **Fimasartan+Amlodipine** | 651900ATB, 652000ATB, 652700ATB, 652100ATB |
| **Fimasartan+Hydrochlorothiazide** | 522000ATB, 526800ATB |
| **Fimasartan+Rosuvastatin** | 655000ATB, 654900ATB, 654800ATB, 654700ATB, 654600ATB |
| **Fosinopril** | 163501ATB, 163502ATB |
| **Hydralazine** | 170701ATB |
| **Imidapril** | 173402ATB, 173401ATB |
| **Irbesartan** | 177301ATB, 177303ATB |
| **Irbesartan+Atorvastatin** | 524000ATB, 524100ATB, 527100ATB, 527000ATB |
| **Irbesartan+Hydrochlorothiazide** | 385700ATB, 385800ATB, 553800ATB |
| **Lacidipine** | 180301ATB, 180302ATB, 180303ATB |
| **Lercanidipine** | 182001ATB, 182002ATB |
| **Lisinopril** | 184501ATB |
| **Lisinopril+Hydrochlorothiazide** | 499200ATB, 499300ATB |
| **Losartan** | 185701ATB, 185702ATB |
| **Losartan+Amlodipine** | 503000ATB, 637400ATB, 513900ATB, 637500ATB, 502700ATB, 637600ATB |
| **Losartan+Hydrochlorothiazide** | 262500ATB, 486900ATB, 378900ATB |
| **Manidipine** | 188001ATB, 188002ATB |
| **Metoprolol** | 194003ATR, 193802ATB, 262400ATR |
| **Metoprolol+Hydrochlorothiazide** | 262600ATB |
| **Metoprolol+felodipine** | 262400ATR |
| **Minoxidil** | 196102ATB |
| **Nadolol** | 198301ATB |
| **Nebivolol** | 489501ATB, 489502ATB, 489503ATB |
| **Nicardipine** | 201003ACR, 201002ATB |
| **Nifedipine** | 201407ACS, 201405ATR, 528201ATR, 201409ATR, 528202ATR, 201401ACS, 201401ATB, 201408ATR |
| **Nimodipine** | 201901ATB, 356202ATR, 356203ATR, 356201ATB, 356202ATB |
| **Nisoldipine** | 356202ATR |
| **Olmesartan** | 468502ATB, 468501ATB, 468503ATB, 520902ATB, 520901ATB |
| **Olmesartan+Amlodipine** | 547800ATB, 632800ATB, 500500ATB, 547700ATB, 629500ATB, 631300ATB, 500600ATB, 547900ATB, 632900ATB, 547600ATB, 548000ATB, 582200ATB, 629600ATB, 633000ATB, 547500ATB, 582400ATB, 629400ATB |
| **Olmesartan+Amlodipine+Hydrochlorothiazide** | 519800ATB, 519700ATB, 520100ATB, 520000ATB, 519900ATB |
| **Olmesartan+Hydrochlorothiazide** | 513600ATB, 489100ATB |
| **Olmesartan+Rosuvastatin** | 644200ATB, 644100ATB, 526900ATB, 526300ATB, 526400ATB, 653200ATB, 526500ATB |
| **Perindopril** | 211301ATB, 501601ATB, 211302ATB, 501602ATB |
| **Perindopril+Indapamide** | 556200ATB |
| **Propranolol** | 219901ATB, 219904ATB, 219906ACR, 219905ACR |
| **Quinapril** | 221901ATB |
| **Ramipril** | 222401ATB, 222402ATB, 222404ATB |
| **Ramipril+Felodipine** | 447100ATB, 447200ATB |
| **Ramipril+Hydrochlorothiazide** | 448600ATB, 448700ATB |
| **Telmisartan** | 378801ATB, 378802ATB, 378803ATB |
| **Telmisartan+Amlodipine** | 521200ATB, 511600ATB, 521300ATB, 511700ATB, 521400ATB, 511500ATB, 644800ATB, 623100ATB |
| **Telmisartan+Hydrochlorothiazide** | 443200ATB, 443300ATB, 502600ATB |
| **Telmisartan+Rosuvastatin** | 631600ATB, 629900ATB, 630000ATB, 631700ATB, 630100ATB, 630200ATB |
| **Temocapril** | 235002ATB |
| **Terazosin** | 235501ATB, 235502ATB, 235503ATB, 616501ATB |
| **Valsartan** | 247103ATB, 247101ATB, 247102ATB, 247104ATB |
| **Valsartan+Amlodipine** | 522600ATB, 492900ATB, 522900ATB, 523200ATB, 522700ATB, 492800ATB, 522800ATB, 523000ATB, 523300ATB, 495800ATB, 523100ATB, 523400ATB |
| **Valsartan+Hydrochlorothiazide** | 356400ATB, 442600ATB |
| **Valsartan+Lercanidipine** | 522200ATB, 522300ATB, 522400ATB |
| **Valsartan+Pitavastatin** | 635000ATB, 635200ATB, 634900ATB, 635100ATB |
| **Valsartan+Rosuvastatin** | 629700ATB, 525000ATB, 525200ATB, 629800ATB, 525100ATB, 525300ATB |
| **Valsartan+Sacubitril** | 651401ATB, 651402ATB, 651403ATB |
| **Verapamil** | 247606ATB, 247607ATB, 247603ATR, 247605ATR, 247601ACR |
| **Zofenopril** | 510401ATB, 510402ATB, 510403ATB |
| **Atorvastatin** | 111501ATB, 111502ATB, 111503ATB, 111504ATB, 502201ATB, 502202ATB, 502203ATB, 502204ATB |
| **Atorvastatin+Amlodipine** | 472300ATB, 472400ATB |
| **Atorvastatin+Ezetimibe** | 633800ATB, 633900ATB, 634800ATB |
| **Fluvastatin** | 162401ACH, 162402ACH, 162403ATR |
| **Lovastatin** | 185801ATB |
| **Pitavastatin** | 470901ATB, 470902ATB, 470903ATB |
| **Pitavastatin+Fenofibrate** | 679300ACH |
| **Pravastatin** | 216601ATB, 216602ATB, 216603ATB, 216604ATB |
| **Rosuvastatin** | 454001ATB, 454002ATD, 454002ATB, 454003ATB, 454003ATD, 454005ATB |
| **Rosuvastatin+Ezetimibe** | 640700ATB, 640800ATB, 640900ATB |
| **Rosuvastatin+Ezetimibe+Telmisartan** | 671400ATB, 671500ATB, 671700ATB |
| **Simvastatin** | 227801ATB, 227802ATB, 227803ATB, 227805ATB, 227806ATB |
| **Aspirin** | 110701ATB, 110702ATB, 110801ATB, 110802ATB, 111001ACE, 111001ATB, 111001ATE, 111002ATE, 111003ACE, 111003ATE |
| **Clopidogrel** | 133201ACR, 133201ATB, 133201ATR, 133202ATB, 133203ATR, 506100ATB |
| **Cilostazol** | 136901ATB, 492501ATB, 495201ATB, 498801ATB, 501501ATB |
| **Ticlopidine** | 498900ATB, 239201ATB, 239202ATB |
| **Aspirin+Bethocarbamol** | 256800ATB |
| **Aspirin+Clopidogrel** | 517900ACH, 517900ACE, 517900ATE, 667500ACE |
| **Aspirin+Dipyridamole** | 489700ACR |

**Table S2. ICD–10 codes used in** Charlson Comorbidity Index

| **Comorbidities** | **Codes** |
| --- | --- |
| **Myocardial infarction** | I21, I22, I252 |
| **Congestive heart failure** | I43, I50, I099, I110, I130, I132, I255, I420, I425–I429, P290 |
| **Peripheral vascular disease** | I70, I71, I731, I738, I739, I771, I790, I792, K551, K558, K559, Z958, Z959 |
| **Cerebrovascular disease** | G45, G46, I60–69, H340 |
| **Dementia** | F00–03, G30, F051, G311 |
| **Chronic pulmonary disease** | J40–47, J60–67, I278–279, J701, J703, J684 |
| **Rheumatologic disease** | M05–06, M32–34, M315, M351, M353, M360 |
| **Peptic ulcer disease** | K25–28 |
| **Mild liver disease** | B18, K73, 74, K700–703, K709, K713–715, K717, K760, K762–764, K768–769, Z944 |
| **DM without complication** | E100–101, E106, E108–111, E116, E118–121, E126, E128–131, E136, E138–141, E146, E148–149 |
| **DM with complication** | E102–105, E107, E112–115, E117, E122–125, E127, E132–135, E137, E142–145, E147 |
| **Hemiplegia or paraplegia** | G81–82, G041, G114, G800, G830–834, G839 |
| **Any malignancy** | C00–26, C30–C34, C37–41, C43, C45–58, C60–76, C81–88, C90–97 |
| **Moderate to severe liver disease** | I850, I859, I864, I982, K704, K711, K721, K729, K765–767 |
| **Metastatic tumor** | C77–80 |
| **AIDS/HIV** | B20–22, B24. |

**Abbreviations**: ICD–10, International Classification of Diseases, 10^th^ revision, Clinical Modification; DM, diabetes mellitus; AIDS/HIV, acquired immune deficiency syndrome/human immunodeficiency virus

**Table S3**. Codes associated with cardiac and cerebrovascular outcomes.

| **Procedure or operation codes** |  |
| --- | --- |
| Percutaneous coronary intervention | M6551, M6552, M6561~M6564, M6571, M6572, M6601, M6602 |
| Coronary artery bypass grafting | O1641, O1642, O1647, OA641, OA642, OA647 |
| **Medical treatment codes** |  |
| Protein C | 635801BIJ |
| Tissue type plasminogen activator | 223501BIJ, 223502BIJ |
| Tenecteplase | 450302BIJ, 450301BIJ |
| Tirofiban | 240201BIJ, 240230BIJ |
| Urokinase | 246401BIJ, 246405BIJ, 246407BIJ, 246404BIJ, 246406BIJ |

**Figure S1. Forest plots of the HR and 95% CI according to subgroups.** (A, B) Univariate Cox regression analysis. (C, D) Multivariate Cox regression analyses. Adjustment was made according to age, sex, underlying cause of end–stage renal disease, Charlson Comorbidity Index score, types of vascular access, hemodialysis vintage, ultrafiltration volume, Kt/V_urea_, hemoglobin, serum albumin, serum creatinine, serum phosphorus, serum calcium, systolic blood pressure, diastolic blood pressure, the use of renin–angiotensin system blockers, statin, ticlopidine, clopidogrel, and aspirin, and myocardial infarction or congestive heart failure.

**Abbreviations:** CI, confidence interval; DM, diabetes mellitus; Group 1, patients without prescription of β–blockers; Group 2, patients with prescription of dialyzable and cardioselective β–blockers; Group 3, patients with prescription of non-dialyzable and non-cardioselective β–blockers; Group 4, patients with prescription of non-dialyzable and cardioselective β–blockers; HR, hazard ratio; HtDs, patients with myocardial infarction or congestive heart failure; Non–HtDs, patients without myocardial infarction and congestive heart failure; Yr, years.

**Figure S2. Kaplan–Meier curves depicting CVE–free survival based on different groups.** The *P*–values for pairwise comparison or trend with log–rank tests are added to the upper right corner of the graph. Abbreviations: CVE, cardiac and cerebrovascular events; Group 1, patients without prescription of β–blockers; Group 2, patients with prescription of dialyzable and cardioselective β–blockers; Group 3, patients with prescription of non-dialyzable and non-cardioselective β–blockers; Group 4, patients with prescription of non-dialyzable and cardioselective β–blockers

**Table S4. Cox regression analyses for mortality in patients with MI, CHF, or atrial fibrillation.**

|  | **Univariate** | | **Multivariate** | |
| --- | --- | --- | --- | --- |
|  | **HR (95% CI)** | ***P*** | **HR (95% CI)** | ***P*** |
| **MI** |  |  |  |  |
| Ref: Group 1 |  |  |  |  |
| Group 2 | 0.93 (0.79–1.09) | 0.348 | 0.94 (0.76–1.17) | 0.590 |
| Group 3 | 0.94 (0.86–1.03) | 0.184 | 0.98 (0.88–1.11) | 0.789 |
| Group 4 | 0.72 (0.72–1.23) | 0.643 | 0.88 (0.62–1.26) | 0.487 |
| Ref: Group 2 |  |  |  |  |
| Group 3 | 1.02 (0.86–1.20) | 0.839 | 1.04 (0.84–1.30) | 0.700 |
| Group 4 | 1.01 (0.75–1.37) | 0.935 | 0.93 (0.62–1.40) | 0.744 |
| Ref: Group 3 |  |  |  |  |
| Group 4 | 1.00 (0.76–1.31) | 0.973 | 0.89 (0.62–1.28) | 0.544 |
| **CHF** |  |  |  |  |
| Ref: Group 1 |  |  |  |  |
| Group 2 | 1.02 (0.94–1.10) | 0.631 | 1.08 (0.98–1.20) | 0.125 |
| Group 3 | 0.93 (0.90–0.98) | 0.002 | 0.97 (0.91–1.02) | 0.198 |
| Group 4 | 0.97 (0.85–1.12) | 0.682 | 0.93 (0.78–1.11) | 0.407 |
| Ref: Group 2 |  |  |  |  |
| Group 3 | 0.92 (0.84–0.99) | 0.040 | 0.89 (0.80–0.99) | 0.031 |
| Group 4 | 0.95 (0.82–1.11) | 0.540 | 0.86 (0.70–1.04) | 0.124 |
| Ref: Group 3 |  |  |  |  |
| Group 4 | 1.04 (0.90–1.20) | 0.588 | 0.96 (0.80–1.15) | 0.664 |
| **Atrial fibrillation** |  |  |  |  |
| Ref: Group 1 |  |  |  |  |
| Group 2 | 1.14 (1.00–1.31) | 0.058 | 1.09 (0.91–1.30) | 0.336 |
| Group 3 | 0.90 (0.82–0.98) | 0.019 | 0.94 (0.84–1.06) | 0.323 |
| Group 4 | 0.89 (0.68–1.15) | 0.372 | 0.85 (0.60–1.21) | 0.362 |
| Ref: Group 2 |  |  |  |  |
| Group 3 | 0.79 (0.68–0.91) | 0.002 | 0.86 (0.72–1.04) | 0.131 |
| Group 4 | 0.78 (0.58–1.04) | 0.086 | 0.78 (0.53–1.14) | 0.199 |
| Ref: Group 3 |  |  |  |  |
| Group 4 | 0.99 (0.76–1.30) | 0.931 | 0.90 (0.63–1.29) | 0.567 |

Multivariate analysis was adjusted for various factors, including age, sex, underlying cause of end-stage renal disease, vascular access, hemodialysis vintage, Charlson Comorbidity Index score, ultrafiltration volume, Kt/V_urea_, hemoglobin, serum albumin, serum creatinine, serum phosphorus, serum calcium, systolic blood pressure, diastolic blood pressure, use of renin-angiotensin system blockers, statin, ticlopidine, clopidogrel, and aspirin, MI or CHF. It was performed using enter mode. MI or CHF as covariate was excluded in multivariate analyses using subgroups with MI or CHF. **Abbreviations**: CHF, congestive heart failure; CI, confidence interval; HR, hazard ratio; MI, myocardial infarction. Group 1, patients without prescription of β–blockers; Group 2, patients with prescription of dialyzable and cardioselective β–blockers; Group 3, patients with prescription of non-dialyzable and non-cardioselective β–blockers; Group 4, patients with prescription of non-dialyzable and cardioselective β–blockers.

**Table S5. Cox regression analyses for CVE**

|  | **Univariate** | | **Multivariate** | |
| --- | --- | --- | --- | --- |
|  | **HR (95% CI)** | ***P*** | **HR (95% CI)** | ***P*** |
| Group |  |  |  |  |
| Ref: Group 1 |  |  |  |  |
| Group 2 | 1.23 (1.10–1.37) | <0.001 | 1.15 (1.00–1.31) | 0.048 |
| Group 3 | 1.09 (1.03–1.15) | 0.002 | 0.99 (0.92–1.05) | 0.682 |
| Group 4 | 1.08 (0.91–1.30) | 0.381 | 1.06 (0.87–1.31) | 0.550 |
| Ref: Group 2 |  |  |  |  |
| Group 3 | 0.89 (0.79–0.99) | 0.035 | 0.86 (0.75–0.99) | 0.034 |
| Group 4 | 0.88 (0.72–1.08) | 0.232 | 0.93 (0.73–1.18) | 0.548 |
| Ref: Group 3 |  |  |  |  |
| Group 4 | 1.00 (0.83–1.19) | 0.966 | 1.08 (0.88–1.33) | 0.469 |

Multivariate analysis was adjusted for several variables including age, sex, underlying cause of end–stage renal disease, vascular access, hemodialysis vintage, Charlson Comorbidity Index score, ultrafiltration volume, Kt/V_urea_, hemoglobin, serum albumin, serum creatinine, serum phosphorus, serum calcium, systolic blood pressure, diastolic blood pressure, use of renin–angiotensin system blockers, statin, ticlopidine, clopidogrel, and aspirin, myocardial infarction or congestive heart failure. It was performed using enter mode. **Abbreviations:** CI, confidence interval; HR, hazard ratio; Group 1, patients without prescription of β–blockers; Group 2, patients with prescription of dialyzable and cardioselective β–blockers; Group 3, patients with prescription of non-dialyzable and non-cardioselective β–blockers; Group 4, patients with prescription of non-dialyzable and cardioselective β–blockers.

**Table S6. Summary of previous studies for the association between the type of β–blockers and clinical outcomes**

| **Author, year** | **Data source** | **Groups and number of patients** | **Main results** |
| --- | --- | --- | --- |
| Wu et al., 2021 [7] | TNHIRD | Bisoprolol (CS + D, n = 9305)  Carvedilol (NCS+ND, n = 11,171) | Bisoprolol was associated with lower mortality and cardiovascular events than carvedilol. |
| Assimon, et al., 2018 [8] | USRDS | Metoprolol (CS+D, n = 17,506)  Carvedilol (NCS+ND, n = 9558) | Metoprolol was associated with lower mortality and cardiovascular events than carvedilol. |
| Shireman et tal., 2016 [9] | USRDS | Atenolol or metoprolol (CS+D)  Carvedilol or labetalol (NCS+ND) | Atenolol or metoprolol was associated with lower mortality and cardiovascular events than carvedilol or labetalol. |
| Tang et al., 2016 [10] | TNHIRD | Bisoprolol (CS+D, n = 629)  Metoprolol (CS+D, n = 63)  Carvedilol (NCS+ND, n = 1008) | In subgroup analysis, there was no significant difference in patient survival among three β–blockers. |
| Wu et al., 2020 [11] | TNHIRD | Atenolol, acebutolol, metoprolol, or bisoprolol (CS+D, n = 15,699)  Betoxolol, carvedilol, or propranolol (NCS or CS +ND, n = 20,904) | D–β–blockers was associated with lower morality and cardiovascular events than ND–β–blockers. |
| Weir et al. 2015 [12] | Healthcare database in Canada | Atenolol, acebutolol, or metoprolol (CS+D, n = 3294)  Bisoprolol (CS+ND) or propranolol (NCS+ND) (n = 3294) | ND–β–blockers was associated with lower mortality and cardiovascular events than D–β–blockers. |

**Abbreviations:** CS, cardioselective; NCS, non-cardioselective; D, dialyzable; ND, non-dialyzable; USRDS, United States Renal Data System; TNHIRD, Taiwan National Health Insurance Research Database
